# Comprehensive Integrated Analyses of Proteins and Metabolites in Equine Seminal Plasma (Horses and Donkeys)

**DOI:** 10.3390/proteomes13030033

**Published:** 2025-07-04

**Authors:** Xin Wen, Gerelchimeg Bou, Qianqian He, Qi Liu, Minna Yi, Hong Ren

**Affiliations:** Inner Mongolia Key Laboratory of Equine Science Research and Technology Innovation, Inner Mongolia Agricultural University, Hohhot 010018, China; wenxin618@imau.edu.cn (X.W.); gerelchimeg@imau.edu.cn (G.B.); qianqianhe202309@163.com (Q.H.); liuqi99967@163.com (Q.L.); yiminna2020@163.com (M.Y.)

**Keywords:** proteomics, metabolomics, seminal plasma, sperm, horse, donkey

## Abstract

Background: The reproductive ability of equine species is a critical component of equine breeding programs, with sperm quality serving as a primary determinant of reproductive success. In this study, we perform an integrative analysis of proteomics and metabolomics in seminal plasma to identify proteins and metabolites associated with sperm quality and reproductive ability in equine species. Methods: We utilized the CEROS instrument to assess the morphology and motility of sperm samples from three horses and three donkeys. Additionally, we statistically analyzed the mating frequency and pregnancy rates in both species. Meanwhile, the 4D-DIA high-throughput proteomic and metabolomic profiling of seminal plasma samples from horses and donkeys revealed a complex landscape of proteins and metabolites. Results: Our findings reveal a certain degree of correlation between seminal plasma proteins and metabolites and sperm quality, as well as overall fertility. Notably, we found that the proteins B3GAT3, XYLT2, CHST14, HS2ST1, GLCE, and HSPG2 in the glycosaminoglycan biosynthesis signaling pathway; the metabolites D-glucose, 4-phosphopantetheine, and 4-hydroxyphenylpyruvic acid in the tyrosine metabolism, starch, and source metabolisms; and pantothenate CoA biosynthesis metabolism present unique characteristics in the seminal plasma of equine species. Conclusions: This comprehensive approach provides new insights into the molecular mechanisms underlying sperm quality and has identified potential proteins and metabolites that could be used to indicate reproduction ability. The findings from this study could be instrumental in developing novel strategies to enhance equine breeding practices and reproductive management. Future research will focus on exploring their potential for clinical application in the equine industry.

## 1. Introduction

Horses and donkeys have played distinct roles throughout human history and in modern society [1,2]. Horses are predominantly utilized in competitive sports, transportation, and recreational activities [3]. Thus, their reproductive capacity is vital for the horse industry’s sustainable development [4]. Interest in donkey reproductive capacity has increased, driven by demand for products such as donkey-hide gelatin, which requires enhancing reproductive efficiency [5]. Understanding the determinants of sperm quality has become a priority in both species as it directly influences fertilization rates [6,7,8]. However, given that our previous analysis of the sperm proteome in horses and donkeys revealed significant differences between the two species [9], this study further compares the proteomic and metabolomic profiles of their seminal plasma. This comparison reveals seminal plasma characteristics and their effects on sperm quality and reproductive capacity. It also aids in elucidating the biological mechanisms underlying sperm quality and reproductive performance in equines.

Seminal plasma, the noncellular component of semen, is a heterogeneous composite fluid composed of secretions from the testis, the epididymis, and the accessory sexual glands [10]. It contains proteins, metabolites, and other biomolecules that surround sperm cells [11]. It provides nutrition, protection, and transportation to sperm. Additionally, it is essential for sperm motility and viability and the ability to fertilize [12,13,14]. Previous studies have demonstrated that seminal plasma protein NPC2 serves as an indicator of bull sperm functionality. Additionally, seminal plasma metabolites, including γ-aminobutyric acid, glutamic acid, and histidine, are closely associated with the physiological properties of semen, thereby offering valuable insights into the investigation of fertility markers in bulls [15,16].

Proteomics refers to the identification and quantification of proteins in biological samples through advanced technological approaches, thereby offering a comprehensive perspective on protein abundance [17,18]. It aids in understanding protein interactions and functions within cells [19]. Moreover, 4D-DIA proteomics can capture all ion signals more comprehensively, furnish more extensive proteome coverage, and provide high-throughput and highly reproducible proteomics data [20]. Proteins in seminal plasma can function as proteins of fertility [21]. For example, Zeng et al. utilized quantitative proteomics to analyze differentially abundant proteins, primarily participating in protein degradation, ATP binding, and energy metabolism, in seminal plasma associated with male fertility [22]. These findings indicate that proteomics related to seminal plasma has been widely applied.

Metabolomics is a methodology for detecting and quantifying metabolites in biological samples through advanced analytical techniques [23]. It is capable of studying the types of, quantities of, and variations in all metabolites within a biological entity, thereby reflecting the physiological and pathological conditions of the organism and revealing its responses to alterations in internal and external environments [24,25]. The seminal plasma metabolome can also be used to identify fertility metabolites [26]. For example, Otero et al. carried out a metabolomics analysis of pig seminal plasma for the purpose of identifying metabolites that can discern fertility within the body [27]. This indicates that metabolomics has been extensively applied in studies related to seminal plasma.

Horse and donkey seminal plasma contains unique proteins and metabolites that regulate reproduction, with these differences linked to their distinct reproductive strategies and adaptations [28,29]. Thus, we hypothesize a potential association between seminal plasma proteins/metabolites and sperm quality/reproductive capacity in horses and donkeys. Furthermore, by systematically comparing the sperm quality and reproductive performances of horses and donkeys, this research can offer a scientific foundation for the conservation of their genetic resources. These insights not only enhance our fundamental knowledge of equine reproductive but also offer new avenues for understanding and improving fertility in equine species.

## 2. Materials and Methods

### 2.1. Experimental Design

**Scheme 1:**
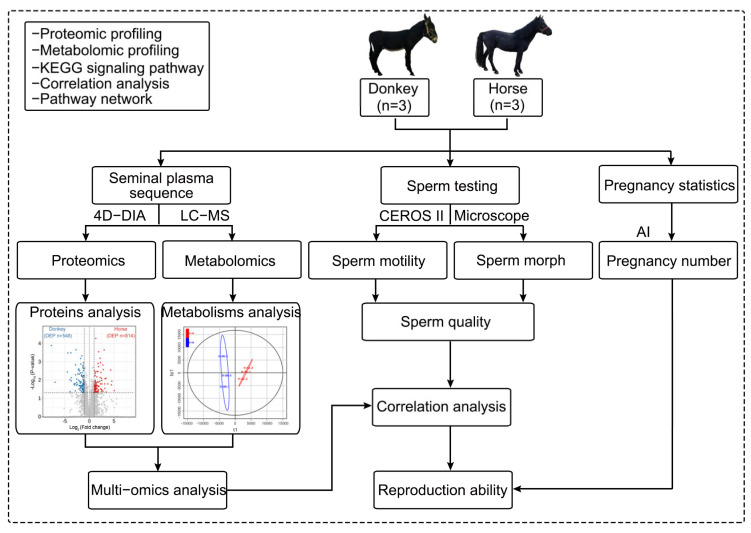
A schematic of the experimental design and workflow. The semen of horses and donkeys was collected and separated to obtain semen plasma, which was used for proteomic and metabolomic sequencing and joint analysis. The remaining semen was utilized to assess sperm and pregnancy statistics, and the results of the seminal plasma sequencing and the sperm quality experiments were subsequently correlated. AI: artificial insemination.

In this study, three stallions and three jacks were selected as the experimental animal samples from the Sport Horse Training Center of Inner Mongolia Agricultural University. The semen of the stallions and jacks was collected for analysis. First, seminal plasma was extracted from the semen of stallions and jacks, and 4D-DIA quantitative proteomics and LC-MS nontargeted metabolomics sequencing were carried out on the extracted seminal plasma. The obtained data were analyzed for differentially abundant proteins and metabolites, as well as for multiomics joint analysis, to obtain a comprehensive landscape of the seminal plasma of horses and donkeys. The remaining semen was subjected to sperm quality testing, including tests for sperm motility and morphology. The sperm samples that met the conditions for artificial insemination experiments were used to statistically analyze the mating and pregnancy statuses of the mare and jenny. Finally, the sequencing results of the seminal plasma were correlated with sperm quality to identify the proteins and metabolites in the seminal plasma related to sperm quality and reproductive ability and their intrinsic connections. A schematic of the experimental design and workflow is shown below (Scheme 1).

### 2.2. Experimental Animals

The experimental animals included 3 male Mongolian horses and 3 male North China donkeys, which originated from the Sport Horse Training Center of Inner Mongolia Agricultural University during the breeding season, which spanned from June to August 2022. In this study, all the horses and donkeys involved were proven breeders. These animals had successfully impregnated mares or jennies and produced offspring through artificial insemination in at least two breeding seasons in the past. Detailed breeding records were collected to ensure that the selected animals had reliable fertility. The average age of these animals was 5 years old, and they were individually housed in comfortable stalls furnished with straw bedding. Standard feed and water were provided throughout the experimental period. All research procedures conformed to relevant regulations and were approved by the Animal Protection Committee of Inner Mongolia Agricultural University.

### 2.3. Seminal Plasma Collection

Individually, stallions and jacks were guided to the artificial mare stand, and artificial sperm collection was carried out after they were sexually primed. Post ejaculation, the semen was promptly centrifuged at 2000× *g* for 10 min to obtain the supernatant. The supernatant was subsequently centrifuged at 1000× *g* for 10 min to remove any remaining sperm and cell debris. Ultimately, 1%*v*/*v* proteinase inhibitor PMSF (ST507, Beyotime, Shanghai, China) was added to the final supernatant. Fresh seminal plasma samples were used for subsequent experiments.

### 2.4. Sperm Assessment

A specific volume of stallion and jack semen samples was collected. The sperm was diluted to a concentration of 5 × 10^7^/mL using the INRA-96^®^ (016441, IMV Technologies, Léger, France) semen extender. The diluted samples were then injected into the Makler counting chamber (Sefi Medical Instruments, Haifa, Israel) of the CEROS II sperm analyzer (Hamilton Thorne, Beverly, MA, USA), and analysis was performed according to the preset parameters of the instrument. The CEROS II instrument is a computer-assisted sperm analysis (CASA) system capable of precisely measuring sperm motility parameters, including velocity and movement trajectory. By adjusting parameter settings, the CEROS II instrument can accurately classify sperm into fast, slow, and non-progressive motility categories, thereby providing reliable data for the assessment of sperm quality. The specific data included sperm morphology (bent tail, coiled tail, DMR, distal droplet, and proximal droplet) and motility (percentages of static, progressive, motile, and slow) and were evaluated via an Olympus Axio Lab.A1 microscope (Olympus, Tokyo, Japan).

### 2.5. Artificial Insemination of Female Livestock

Artificial insemination was carried out immediately after sample collection. Before insemination, strict follicular monitoring was conducted on the mares and jennies. When the dominant follicle reached a diameter of over 35 mm, an intramuscular injection of 2000 IU HCG (Ningbo Hormone Factory, Ningbo, China) was administered. Artificial insemination was performed 24–36 h post injection. Following the disinfection of the vaginal openings of the horse and donkey, artificial insemination was carried out by manually ascertaining the location of the uterus. The semen was subsequently injected into a syringe, and an artificial insemination tube (CASA, Paris, France) was delicately inserted into the uterine cavity, with the semen being injected through the syringe. After the semen was injected into the uterus, the artificial insemination tube was withdrawn, and gentle massage was administered to the cervix to prevent the backflow of the semen. Then, 48 h after insemination, we monitored for ovulation; if ovulation had not occurred, insemination was repeated until successful ovulation was confirmed. Between 14 and 18 days post-insemination, the mare’s or jenny’s uterus was examined using a veterinary B-mode ultrasound device to assess endometrial thickness and gestational sac development. Pregnancy was confirmed if a non-echoic gestational sac with a diameter of ≥10 mm or fetal heart activity was identified. The effective sperm count in each insemination dose typically ranged from 100 million to 300 million sperm. The specific quantity was adjusted based on factors such as the quality of the stallion or jack semen, the reproductive history of the mare or jenny, and the current breeding season. Semen samples were diluted with INRA-96^®^ to extend the sperm survival time and maintain their vitality and fertilization ability. The dilution ratio was adjusted according to initial semen quality and sperm density, typically ranging from 1:3 to 1:5, to ensure the optimal function of the diluted sperm. In this study, due to the need for the artificial insemination of a large number of mares and jennies using fresh semen for each mating, the stallions and jacks used as experimental animals required multiple ejaculation (twice a week). Meanwhile, the artificial inseminations were performed by the same person to ensure consistency in the experiment. Notably, stallions were mated with mares, and jacks with jennies.

### 2.6. Proteomics and LC-MS/MS

The 4D-DIA protein sequencing work strictly followed the standard workflow: protein precipitation, reduction, alkylation, and trypsin digestion. Specifically, 200 μL of whole seminal plasma samples were individually collected from stallions and jacks, and the protein concentration was quantified using the BCA assay kit (P0012S, Beyotime, Shanghai, China). Protein solutions were prepared by taking equal amounts based on protein concentration and adjusting the volume to 200 μL with 8 M urea. The samples were then reduced with 10 mM DTT at 37 °C for 45 min, followed by alkylation with 50 mM iodoacetamide in the dark at room temperature for 15 min. Subsequently, four volumes of pre-cooled acetone relative to the protein solution were added, and the mixture was incubated at −120 °C for 2 h to precipitate the proteins. After centrifugation, the protein pellets were air-dried and resuspended in 200 μL of 25 mM ammonium bicarbonate containing 3 μL of trypsin (V5280, Promega, Madison, WI, USA). Digestion was performed overnight at 37 °C. Following digestion, the resulting peptides were desalted using a C18 column, concentrated via vacuum centrifugation, and finally redissolved in 0.1% (*v*/*v*) formic acid. The samples were separated using a NanoElute UHPLC (Bruker, Bremen, Germany) system operating at a nanoliter flow rate. Mobile phase A consisted of 0.1% formic acid in water, while mobile phase B consisted of 0.1% formic acid in acetonitrile (100% acetonitrile). Samples were automatically injected onto an analytical column (aur 2-25075 c 18 a, IonOpticks, Fitzroy, Australia; 25 cm × 75 μm, C18 packing material, 1.6 μm particle size) by an autosampler. The column temperature was maintained at 50 °C using an integrated column oven. The injection volume was 200 ng, the flow rate was set to 300 nL/min, and the total gradient duration was 40 min. The liquid chromatographic gradient was as follows: 0–25 min, with the B phase increased from 2% to 22%; 25–30 min, with the B phase linearly increased from 22% to 35%; 30–35 min, with the B phase linearly increased from 35% to 80%; 35–40 min, with the B phase remained constant at 80%. First, the mixed samples were separated by chromatography and then the mass spectrometry data were collected using the ddaPASEF mode of the timsTOF Pro2 mass spectrometer to establish the appropriate acquisition windows for the diaPASEF acquisition method. The effective gradient for analysis was 40 min, with positive ion detection, a parent ion scanning range of 100–1700 *m*/*z*, an ion mobility range of 0.85–1.3 V·s, an ion accumulation and release time of 100 ms, an ion utilization rate of nearly 100%, a capillary voltage of 1500 V, a drying gas flow rate of 3 L/min, and a drying temperature of 180 °C. The parameters for the DDA-PASEF acquisition mode were 4 MS/MS scans (total cycle time of 0.53 s), a charge range of 0–5, a dynamic exclusion time of 0.4 min, an ion target intensity of 10,000, an ion intensity threshold of 1500, a collision energy linearly increasing with ion mobility, a collision energy of 27 eV at 1/of 0.85 V·s/, and an energy of 45 eV at 1/of 1.3. When *m*/*z* < 700, the quadrupole isolation width was set to 2 Th; when *m*/*z* > 800, it was set to 3 Th. The parameters for the diaPASEF acquisition mode were a mass range approximately 400–1200,a mobility range of 0.85–1.3 V·s/, a mass width of 25 Da, a mass overlap of 0.1, 24 mass steps per cycle of 24, and 2 mobility windows, totaling 48 acquisition windows. The average acquisition cycle was 1.17 s.

### 2.7. Proteomic Data Analysis

In this study, we established the criterion of at least two unique peptide matches as a stringent threshold for inferring the presence of an amino acid sequence. Specifically, a protein was deemed reliably identified only if at least two distinct peptides corresponding to it exhibited high-confidence matches with theoretical peptide sequences based on mass spectrometry data. The DIA mass spectrometry data in this project were analyzed using the DIA-NN software (v1.8.1). The search parameters included the following databases: uniprotkb_proteome_UP000002281_Equus_caballus_20231115.fasta (comprising 69,434 sequences) and uniprotkb_proteome_UP000694387_Equus_asinus_20231115.fasta (comprising 48,973 sequences). Deep learning-based parameters were employed to predict a spectral library, while the MBR option was enabled to generate a spectral library from the DIA data and re-analyze the data for protein quantification. Following the completion of protein quantification by the database search software, the intensity values of each protein across different samples as provided in the search results had to be extracted. Subsequently, centralization transformation was applied to perform intra-sample standardization, yielding the relative quantification values (R) of proteins across different samples. The calculation formula was as follows: $R_{ij}=I_{ij}/Median(I_{i})$. Subsequently, differential quantification analysis was carried out on the proteins, and differentially abundant proteins were selected from each comparison group. A global FDR ≤ 5% (false discovery rate control at both the peptide and protein levels) was adopted, and we applied the adjusted p value with a significance threshold of 0.05. In this study, we used internal standards of known concentrations, such as MWS04187, for the quantification of metabolites. The missing values were mainly distributed in the secondary functional proteins. The optimal K = 5 could be determined through leave-one-out cross-validation to balance the imputation accuracy and noise resistance. Comprehensive functional annotation was subsequently conducted on the identified proteins and the differential proteins in each comparison group. This encompassed detailed annotations such as Gene Ontology (GO) and Kyoto Encyclopedia of Genes and Genomes (KEGG) pathway annotations.

### 2.8. Metabolomics and LC-MS

We collected 200 μL of whole seminal plasma samples from stallions and jacks individually, then transferred them into the corresponding sample vials for subsequent analysis. Chromatographic separation was achieved using a Waters ACQUITY Premier HSS T3 Column (Waters Corporation, Milford, MA, USA. 1.8 µm particle size, 2.1 mm inner diameter, and 100 mm length), which provided high column efficiency and excellent separation selectivity for sample analysis. The mobile phase system consisted of two components: mobile phase A (0.1% formic acid in water) and mobile phase B (0.1% formic acid in acetonitrile). The inclusion of formic acid enhanced the ionization efficiency and improved peak shape during mass spectrometry detection. The column temperature was maintained at 40 °C to ensure stable and reproducible chromatographic performance. The flow rate was set at 0.4 mL/min, with an injection volume of 4 μL. During the gradient elution process, the proportion of mobile phases was adjusted as follows: at 0.0 min, 95% A and 5% B; at 2.0 min, 80% A and 20% B; at 5.0 min, 40% A and 60% B; at 6.0 min, 1% A and 99% B; this was maintained until 7.5 min. Subsequently, the mobile phase ratio rapidly returned to the initial conditions (95% A and 5% B) at 7.6 min and remained constant until 10.0 min, ensuring the effective separation of various metabolites in the sample. Mass spectrometry analysis was performed using an AB TripleTOF 6600 system (SCIEX, Framingham, MA, USA). Data acquisition was carried out in both positive ion mode (ESI+) and negative ion mode (ESI−), with a total acquisition time of 10 min for each mode. In ESI+ mode, the ionization voltage was set to 5000 V and the ion source temperature was set to 550 °C, and the pressures of the spray gas, auxiliary heating gas, and curtain gas were adjusted to 50 psi, 60 psi, and 35 psi, respectively. The declustering potential, MS1 collision energy, MS2 collision energy, and collision energy step size were configured as 60 V, 10 V, 30 V, and 15 V, respectively. The mass range for MS1 and MS2 was set to 50–1000 Da and 25–1000 Da, respectively, with acquisition times of 0.2 s and 0.04 s. A total of 18 candidate ions were selected for analysis. In ESI− mode, the ionization voltage was set to −4000 V, and the ion source temperature was adjusted to 450 °C. All gas pressure parameters remained consistent with those in ESI+ mode. The declustering potential, MS1 collision energy, MS2 collision energy, and collision energy step size were configured as -60 V, −10 V, −30 V, and 15 V, respectively. The mass range, acquisition time, and number of candidate ions for MS1 and MS2 were identical to those in ESI+ mode. These parameter settings effectively maximized the ionization efficiency and detection sensitivity of metabolites, thereby enabling comprehensive and accurate analysis of complex metabolite mixtures. The other specific parameter conditions were set in accordance with Wuhan MetWare Biotechnology Co., Ltd. (Wuhan, Hubei, China).

### 2.9. Metabolomic Data Analysis

This study adopted a nontargeted metabolomics analysis strategy. The data preprocessing workflow comprised peak extraction, retention time correction, peak alignment, missing value handling, normalization, and data scaling to ensure data accuracy and comparability. The specific steps were as follows: The raw mass spectrometry data (raw format) were converted into mzXML format using ProteoWizard (v3.0) for subsequent software compatibility. Peak extraction was performed using the CentWave algorithm in XCMS (R package, v3.18.3). The mass-to-charge ratio (*m*/*z*) error tolerance was set at ±5 ppm (positive mode) or ±10 ppm (negative mode), with a peak width range of 5–30 s and a signal-to-noise ratio threshold of 5. Retention time drift across samples was corrected globally using the obiwarp algorithm in XCMS, with a correction window of ±0.2 min. Peak alignment was achieved using the group function in XCMS combined with the Dynamic Time Warping (DTW) algorithm to match identical metabolite peaks across different samples, generating a matrix containing retention time, *m*/*z* values, and peak areas. Metabolite peaks missing in ≥50% of the samples were removed to minimize noise interference. Missing values retained were imputed using the K-Nearest Neighbor (KNN, K = 5) algorithm based on the Euclidean distance between samples. Background signals from blank samples (e.g., extraction solvents) were subtracted; if the peak area of a metabolite in the blank exceeded 10% of the sample mean, it was considered contaminated and excluded. Batch effects and instrument drift-induced variations in peak areas were corrected using the Support Vector Regression (SVR) algorithm, with quality control (QC) samples used as references. Differences in metabolite concentration ranges that could affect statistical analysis were eliminated. The corrected peak areas were Pareto-scaled using a formula, which retained the trend of the original data while reducing the weight of high-abundance metabolites, making it suitable for multivariate statistical analyses such as principal component analysis (PCA) and orthogonal partial least squares discriminant analysis (OPLS-DA). A mixture of all biological samples served as QC samples, with one QC sample inserted after every 10 biological samples to monitor analytical stability. Repeatability was assessed by the coefficient of variation (CV) of the peak areas of QC samples, requiring that >85% of metabolites had CV < 0.3. In the PCA, QC sample points clustered tightly and were separated from biological sample groups, indicating that preprocessing effectively minimized technical variability. In metabolomics data analysis, we applied the adjusted *p* value with a significance threshold of 0.05. The validation methods for PLS-DA models were as follows: The predictive parameters of the OPLS-DA evaluation model included R2X, R2Y, and Q2. Among them, R2X and R2Y represented the explanatory power of the established model for the X and Y matrices, respectively, and Q2 indicated the predictive ability of the model. The closer these three indicators were to 1, the more stable and reliable the model was. A model was considered effective when Q2 > 0.5 and excellent when Q2 > 0.9. In total, 200 random permutation and combination experiments were conducted on the data. If the p of Q2 was 0.02, this indicated that there were 4 random grouping models with better predictive ability than this OPLS-DA model in this permutation test. If the p of R2Y was 0.545, this indicated that there were 109 random grouping models with better explanatory power for the Y matrix than this OPLS-DA model in this permutation test. Generally, a model was considered optimal when *p* < 0.05.

### 2.10. Combined Proteomics and Metabolomic Analysis

For the combined proteomics and metabolomic analysis, differentially abundant proteins and differentially abundant metabolites were mapped onto pathways by using the KEGG database. Enrichment analysis was performed with R to combine the KEGG annotation and enrichment results, providing a comprehensive comprehension of the affected signaling pathways.

### 2.11. PPI and Correlation Matrix

Protein–protein interactions (PPIs) were retrieved from the STRING database (version 11.5, https://cn.string-db.org/, access date: 7 November 2024) using the STRING Cytoscape plugin with a confidence threshold of 0.7, specific to equine species (Equus caballus for horses and Equus asinus for donkeys). The resulting PPI network was visualized using Cytoscape v3.9.1. For correlation analysis, a correlation matrix was generated by calculating the Pearson correlation coefficients between the identified seminal plasma proteins and metabolites in horses and donkeys and their sperm motility parameters, and multiple comparison corrections were performed using the Benjamini–Hochberg method. A false discovery rate (FDR) < 0.05 was set as the significance threshold.

### 2.12. Statistical Analysis

The results were analyzed and visualized using GraphPad Prism 9.0 software (GraphPad Prism, La Jolla, CA, USA) and validated through one-way ANOVA, followed by Tukey’s post hoc test for multi-group comparisons and Student’s *t* test for two-group comparison. The results are presented as the means ± standard deviations (SDs), with the significance level set at *p* < 0.05. All the experiments were carried out with three biological replicates. Normality tests were conducted using the Shapiro–Wilk test (suitable for small samples, *n* ≤ 50). The results indicated that most of the indicators did follow a normal distribution (*p* > 0.05). NS indicates no significant difference (*p* > 0.05), a single asterisk (*) indicates a statistically significant difference (*p* < 0.05), two asterisks (**) indicate a highly statistically significant difference (*p* < 0.01), and three asterisks (***) indicate a highly statistically significant difference (*p* < 0.001).

## 3. Results

### 3.1. Testing of Horse and Donkey Sperm Quality

In this study, while performing sequencing analysis, we examined the morphological and motility characteristics of horse and donkey sperm. The findings indicated that the rates of static and slow sperm in horses were significantly greater than those in donkeys (*p* < 0.05). Conversely, regarding the progressive and motile indicators of horses and donkeys, the progressive sperm rate of horses was markedly lower than that of donkeys (35.07 vs. 71.57%, *p* < 0.05), and the motile sperm rate of horses was also significantly lower than that of donkeys (68.37 vs. 88.57%, *p* < 0.05). Regarding the sperm morphology of horses and donkeys, there was no statistically significant disparity between them, either in bent-tail sperm or coiled-tail sperm. The proportion of normal morph sperm was 77.14% in horses and 91.87% in donkeys (Table 1). Overall, the sperm motility and normal sperm morphology, as assessed by the computer-aided sperm analysis system, were both greater than 65%.

### 3.2. Testing of Horse and Donkey Fertility

After we tested the quality of the sperm from stallions and jacks and ascertained that they met the established criteria, we carried out subsequent mating experiments. The findings indicated that the three stallions mated with 37, 29, and 35 mares, with 25, 21, and 23 pregnancies, respectively, resulting in an average pregnancy rate of approximately 69%. Moreover, the three jacks mated with 6, 40, and 30 jennies, with the number of pregnancies being 4, 30, and 23, respectively, leading to an average pregnancy rate of approximately 73%. The statistical results revealed that the pregnancy rates of both horses and donkeys were approximately 70% (Table 2). This outcome is consistent with the majority of the current data, suggesting that the horses and donkeys involved in this study have normal reproductive capacity.

### 3.3. Proteomics Analysis of Horse and Donkey Seminal Plasma

**Figure 1:**
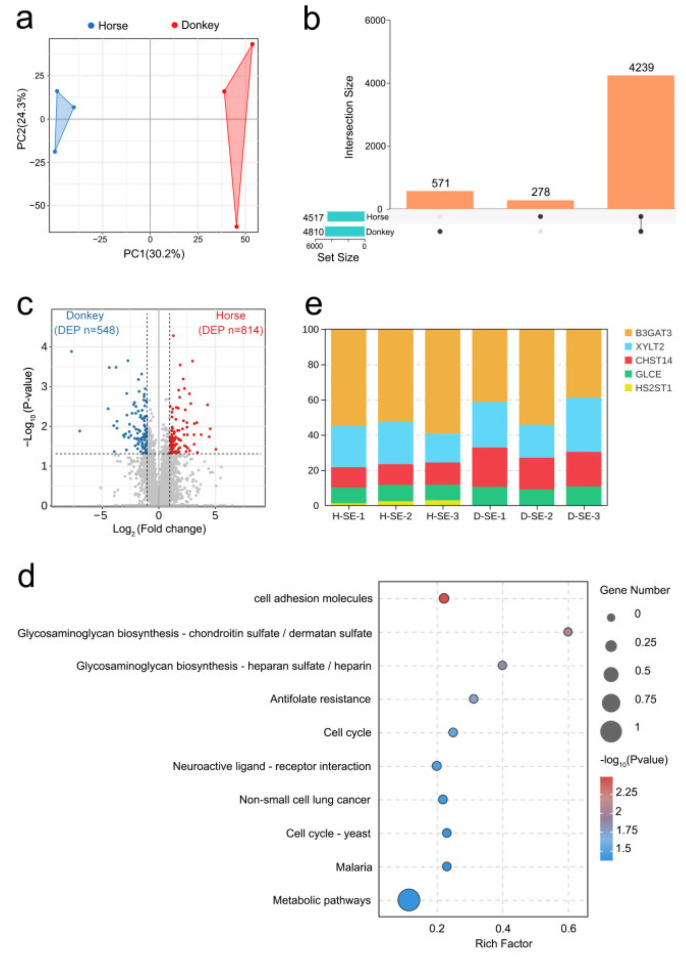
A proteomic analysis of horse and donkey seminal plasma. (**a**) A PCA plot showing the distinct clusters of proteins within the seminal plasma of horses and donkeys. The blue triangles represent samples of horses, and the red triangles represent samples of donkeys. (**b**) UpSet plot revealing the common and specific abundances of proteins in the seminal plasma of horses and donkeys. (**c**) A volcano plot demonstrating the differentially abundant proteins in the seminal plasma of horses and donkeys, where the donkey proteins and horse proteins are represented by blue and red dots, respectively. (**d**) A bubble chart showing the top 10 KEGG signaling pathways enriched with differentially abundant proteins in the seminal plasma of horses and donkeys. (**e**) A stacked bar chart showing the percentages of B3GAT3, XYLT2, CHST14, GLCE, and HS2ST1.

This study employed the most recent quantitative proteomics sequencing technique (4D-DIA) to detect the protein composition and abundance in seminal plasma samples from horses and donkeys. Principal component analysis (PCA) revealed significant disparities in the protein composition of seminal plasma between horses and donkeys (Figure 1a). An UpSet plot revealed the protein abundance in seminal plasma from horses and donkeys. Specifically, 4239 proteins were coexpressed in both horse and donkey seminal plasma, 278 proteins were specifically expressed in horse seminal plasma, and 571 proteins were specifically expressed in donkey seminal plasma (Figure 1b). Circos plots revealed the cellular localization of these proteins in horse and donkey seminal plasma, with 30% localized in the cytoplasm, 22% in the nucleus, 12% in the mitochondria, and 11% in the plasma membrane (Figure S1a). A comprehensive analysis of the proteomics sequencing data for the seminal plasma of donkeys and horses revealed that 548 proteins (blue dots) in donkey seminal plasma presented lower values than the corresponding protein abundance in horse seminal plasma. In contrast, 814 proteins (red dots) in horse seminal plasma presented higher than those in donkey seminal plasma (Figure 1c). These proteins were subsequently annotated and classified based on GO enrichment. The biological processes associated with the differentially abundant proteins were enriched primarily in cellular processes, single-organism processes, and metabolic processes. The enriched molecular functions were closely related to binding, catalytic activity, and molecular function regulation. The enriched cellular components were mainly concentrated in cell parts, cells, and organelles (Figure S1b). Furthermore, the differentially abundant proteins were analyzed using the KEGG database. The results indicated that the proteins were enriched primarily in cell adhesion molecules via glycosaminoglycan biosynthesis: chondroitin sulfate/dermatan sulfate and heparan sulfate/heparin. It was also found that the rich factor was greatest in glycosaminoglycan biosynthesis: chondroitin sulfate/dermatan sulfate and heparan sulfate/heparin; thus, our focus was placed on the glycosaminoglycan biosynthesis signaling pathway (Figure 1d). All the proteins in this signaling pathway were identified, namely B3GAT3, GLCE, CHST14, HS2ST1, and XYLT2 (Figure S1c). Among the five differentially abundant proteins, B3GAT3 had the highest proportion, followed by XYLT2 and CHST14, whereas GLCE and HS2ST1 had the lowest proportions (Figure 1e). The abundances of these proteins in the seminal plasma of horses and donkeys were subsequently analyzed, and the protein abundances in horse seminal plasma were greater than those in donkey seminal plasma for all these proteins (Figure S1d–h). These results on the composition and abundance characteristics of proteins in horse and donkey seminal plasma offer a detailed proteomic profile for equine seminal plasma.

### 3.4. Metabolomics Analysis of Horse and Donkey Seminal Plasma

**Figure 2:**
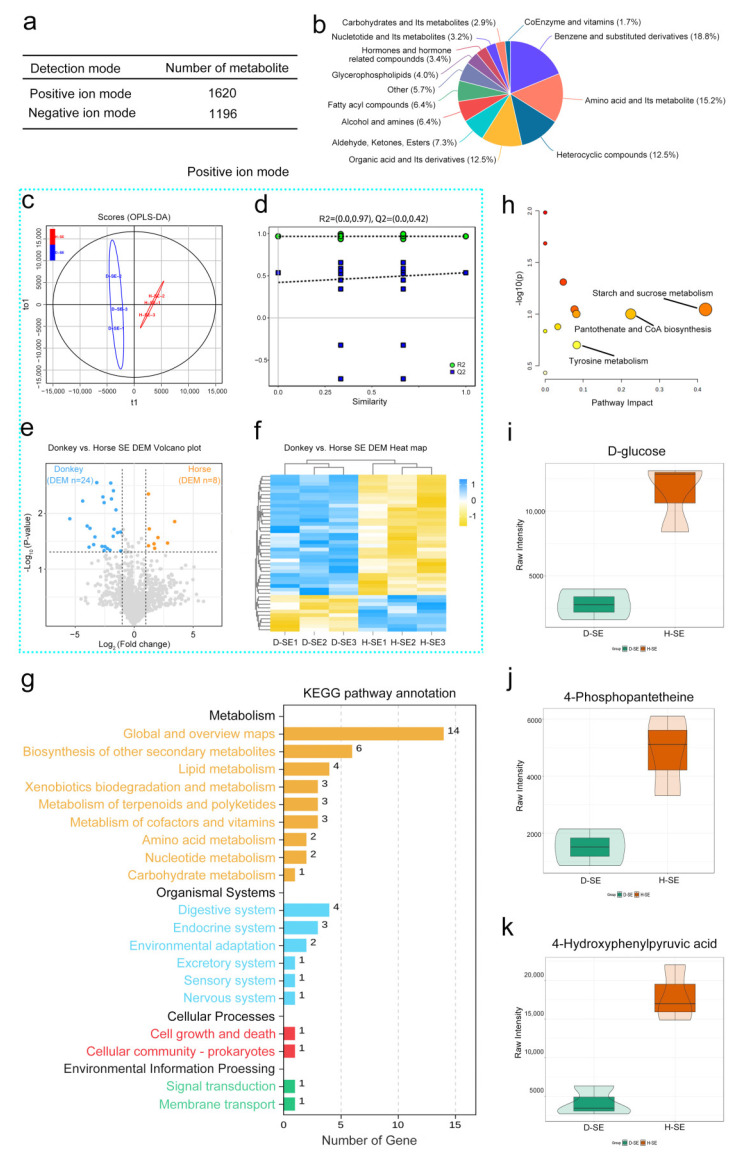
A metabolomics analysis of horse and donkey seminal plasma. (**a**) Counts of metabolites identified in positive and negative ion modes. (**b**) The proportions of identified metabolites within each chemical classification. (**c**) The OPLS–DA of metabolomic data for seminal plasma of donkeys and horses in positive ion mode. (**d**) A diagram of model verification in positive ion mode. (**e**) A volcano plot of differentially abundant metabolites between the seminal plasma of a donkey and a horse in positive ion mode. (**f**) A heatmap of metabolites between the seminal plasma of a donkey and a horse in positive ion mode. The blue dashed box represents the data in the positive mode. (**g**) The enriched KEGG pathways of differentially abundant metabolites between the seminal plasma of donkeys and horses. (**h**) MetPA analysis of key metabolites. (**i**) The abundances of D-glucose in the seminal plasma of horses and donkeys. (**j**) The abundances of 4-phosphopantetheine in the seminal plasma of horses and donkeys. (**k**) The abundances of 4-hydroxyphenylpyruvic acid in the seminal plasma of horses and donkeys. The data are presented as the means ± SDs. *p* < 0.05 was considered to indicate a statistically significant difference.

To identify the characteristic metabolites of horse and donkey seminal plasma, we carried out untargeted metabolomics sequencing. A total of 2816 metabolites were identified, among which 1620 were detected in positive ion mode and 1196 in negative ion mode (Figure 2a). These included benzene and substituted derivatives, amino acids and their metabolites, heterocyclic compounds, organic acids and their derivatives, and other compounds (Figure 2b). In positive ion mode, the OPLS-DA scores of horse and donkey seminal plasma samples were significantly separated into two clusters (Figure 2c). The value of R2 was equal to 0.97, and that of Q2 was equal to 0.42 (a predictive ability parameter, >0.4 indicates a reliable model). Additionally, the 200 exchangeability tests showed that the intercept of the regression line for the random model Q2 was less than 0 on the y-axis, indicating that the model had predictive power (Figure 2d). With a log2 FC > 1.5, 24 differentially abundant metabolites were downregulated in donkey seminal plasma, and 8 differentially abundant metabolites were upregulated in horse seminal plasma (Figure 2e). The data are presented through a heatmap (Figure 2f). In negative ion mode, a distinct separation was observed in the OPLS-DA scores between donkey and horse seminal plasma (Figure S2a). The R2 value was 0.97 and the Q2 value was 0.42, indicating a more precise predictive model (Figure S2b). Under the same differentially abundant fold change, 19 metabolites were downregulated in donkey seminal plasma and 6 metabolites were upregulated in horse seminal plasma (Figure S2c). This is displayed through a heatmap in Figure S2d. KEGG analysis revealed that all of the differentially abundant metabolites were enriched in global and overview maps via the biosynthesis of other secondary metabolites, lipid metabolism, xenobiotic biodegradation and metabolism, other metabolism signaling pathways, protein digestion and absorption, lactose metabolism, the pentose phosphate pathway, and the ubiquitin-mediated pathway (Figure 2g). Upon further analysis of the metabolic pathways, three metabolic pathways, namely starch and sucrose metabolism, pantothenate and CoA biosynthesis, and tyrosine metabolism, were identified (Figure 2h). In starch and sucrose metabolism, the metabolite D-glucose is the characteristic metabolite. In pantothenate and CoA biosynthesis, the metabolite 4-phosphopantetheine is the characteristic metabolite. In tyrosine metabolism, the metabolite 4-hydroxyphenylpyruvic acid is the characteristic metabolite. Moreover, the abundance of the metabolites D-glucose, 4-phosphopantetheine, and 4-hydroxyphenylpyruvic acid in horse seminal plasma were significantly greater than those in donkey seminal plasma (Figure 2i–k). These results on the compositions and abundance characteristics of metabolites in horse and donkey seminal plasma offer a detailed metabolomics profile for equine seminal plasma.

### 3.5. Integrated Proteomics and Metabolomics Analysis

**Figure 3:**
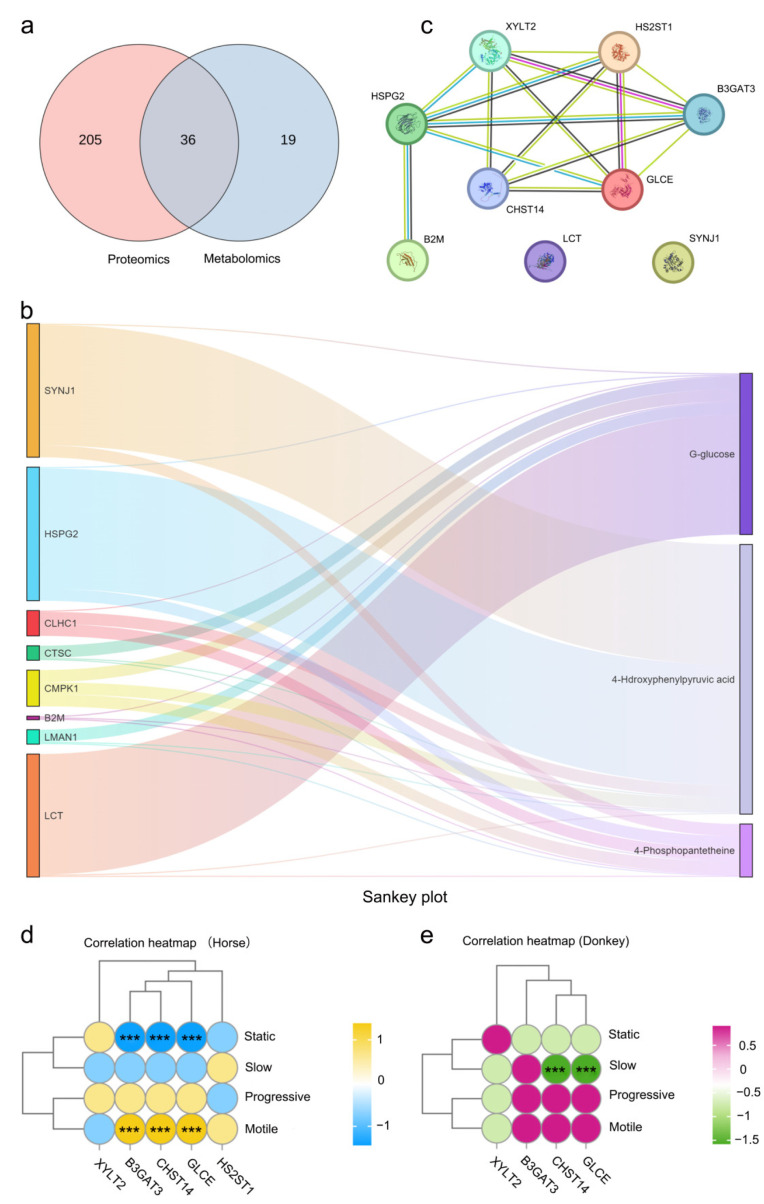
Integrated proteomics and metabolomics analysis. (**a**) A Venn diagram depicting the quantity of pathways analyzed through the joint analysis of proteomics and metabolomics. (**b**) A sunburst diagram revealing the intensity of correlation between differential proteins and metabolites. (**c**) A PPI network in donkey and horse seminal plasma. (**d**) The correlation between the sperm motility of horses and the key proteins in horse seminal plasma. (**e**) The correlation between the sperm motility of donkeys and the key proteins in donkey seminal plasma. (***) indicate a highly statistically significant difference (*p* < 0.001).

An integrated analysis of the differential proteins and metabolites between the seminal plasma of donkeys and horses was carried out to further evaluate the proteins and metabolites for the sperm quality and reproductive ability of equine species. Thirty-six KEGG pathways, including cholesterol metabolism, glycolysis/gluconeogenesis, tyrosine metabolism, starch and sucrose metabolism, glycerophospholipid metabolism, the FoxO signaling pathway, the AMPK signaling pathway, and the biosynthesis of amino acids, were identified through both metabolomic and proteomic analyses (Figure 3a and Figure S3a). Correlation heatmap clustering analysis objectively reflected the abundance patterns of differential proteins and metabolites, grouping similar abundance patterns involving similar biological processes into the same cluster and revealing significantly positive or negative correlations between them (Figure S3b). Further studies revealed that the three key metabolites identified in the metabolome, i.e., D-glucose, 4-phosphopantetheine, and 4-hydroxyphenylpyruvic acid, were significantly correlated with SYNJ1, HSPG2, CLHC1, CTSC, CMPK1, B2M, LMAN1, and LCT in the proteome (Figure 3b). We performed statistical analyses on the strongly correlated SYNJ1, LCT, and HSPG2 proteins, as well as the weakly correlated B2M protein. The results indicated that the relative abundances of these proteins in horse seminal plasma were significantly greater than those in donkey seminal plasma (Figure S3c–f). We subsequently conducted protein-protein interaction (PPI) analysis on SYNJ1, LCT, HSPG2, and B2M proteins with the key proteins B3GAT3, XYLT2, CHST14, HS2ST1, and GLCE identified via proteomic analysis, and the results revealed that SYNJ1, LCT, and B2M had no interactions with B3GAT3, XYLT2, CHST14, HS2ST1, or GLCE proteins, whereas HSPG2 had good interactions with any of these key proteins. Therefore, we hypothesize that the HSPG2 protein may be a key bridge protein among the seminal plasma proteins and metabolites of horses and donkeys (Figure 3c). We assessed the correlation between the quality of sperm (static, slow, progressive, and motile) in the seminal plasma of horses and donkeys and the key proteins B3GAT3, XYLT2, CHST14, HS2ST1, and GLCE. The matrix revealed that the B3GAT3, CHST14, and GLCE proteins in horse seminal plasma were positively associated with the motility index of horse sperm and negatively related to the static index (*p* < 0.05) (Figure 3d). Conversely, the CHST14 and GLCE proteins in donkey seminal plasma were negatively correlated with the slow index of donkey sperm (*p* < 0.05) (Figure 3e). This finding indicates that the 4D-DIA quantitative protein and metabolite profiling of horse and donkey seminal plasma, in conjunction with their combined analysis, is capable of identifying key proteins and metabolites related to horse and donkey sperm motility, facilitating the search for potential protein or metabolites that indicate the sperm quality and the reproductive capacity of equine species.

## 4. Discussion

The integrated analysis of proteomics and metabolomics in seminal plasma has offers novel insights into the complex mechanisms underlying sperm quality and reproductive capacity in equine species. This research systematically characterizes the molecular constituents of seminal plasma and their potential roles in regulating sperm function and overall reproductive success in horses.

Sperm quality assessments revealed distinct motility patterns between horses and donkeys: horse sperm exhibited significantly higher static and slow motility rates but lower progressive motility compared to donkey sperm. These results align with prior reports, such as Gobato’s finding of ~80% donkey sperm motility and Ashlee J Medica’s observation of ~70% horse sperm motility, consistent with our findings [9,30,31]. Gacem’s conclusion that donkey sperm displays faster and more linear movement further supports these interspecies differences [32]. In sperm morphology, no significant differences were detected in normal morphology rates or frequencies of bent/coiled tails between species, corroborating Gacem’s staining-based results showing no abnormal morphological outcomes in equine sperm [33].

This study assessed equine reproductive capacity using sperm-qualified stallions and jacks via artificial insemination. Stallion pregnancy outcomes aligned with Aitken’s predictions from thoroughbred seminal plasma analysis: 103 of 143 mated mares became pregnant (72%) [34]. In contrast, the donkey pregnancy rate (73%) significantly exceeded Diana Fanelli’s reported 27.1%, but aligned with Umberto Tosi’s 70% rate [35]. These disparities may stem from factors like mare status, breeding techniques, or environment, though high-quality sperm maintains relatively high reproductive success. As sperm functions within the seminal plasma milieu, seminal plasma analysis can evaluate sperm quality and predict pregnancy rates in equine species.

This study used 4D-DIA quantitative proteomics to compare seminal plasma proteins between horses and donkeys, revealing species-specific compositional variations linked to reproductive biology. Differentially abundant proteins were significantly enriched in the glycosaminoglycan (GAG) biosynthesis pathway. GAGs bind/transport bioactive molecules (e.g., amino acids, hormones). GAGs bind/transport bioactive molecules (e.g., amino acids, hormones) and protect sperm from environmental stress, maintain membrane integrity, and enhance survival [36,37]. In seminal plasma, GAGs form proteoglycans with proteins to influence sperm motility, morphology, and fertilization capacity [38]. They also regulate epididymal ion concentration and osmotic pressure during sperm maturation/storage, interact with sperm surface receptors to modulate motility and oocyte recognition, and contribute to sperm tail dynamics and zona pellucida penetration [39,40,41]. While these findings highlight the GAG pathway’s role in sperm maturation, motility, and fertilization, specific mechanisms require further investigation. Brzozowska et al. similarly emphasized GAGs and ions as critical for reproductive assessment in canines [42]. This crucial signaling pathway includes a variety of important proteins, among which B3GAT3 is an enzyme involved in the synthesis of sugar chains. In sperm, the B3GAT3 protein may influence sperm motility. Studies have indicated that the abundance of the B3GAT3 protein is associated with sperm viability, motility speed, and morphology [43]. The CHST14 protein is a heparan sulfate acetyltransferase that is involved in the synthesis of the heparan sulfate (HS) chain [44]. With respect to sperm motility, the CHST14 protein might influence the structure and function of the HS chain, thereby affecting sperm motility [45]. Studies have demonstrated that heparan sulfate plays a crucial role in the interaction between sperm and oocytes, potentially by binding to specific receptors on the surface of sperm and participating in the processes of sperm recognition, adhesion, and penetration [46]. The GLCE protein is an enzyme that plays an important role in sperm motility. Studies have indicated that the GLCE protein regulates the structure and function of the sperm tail, and its abnormal abundance might lead to a decrease in sperm motility, thereby affecting male fertility [47]. However, this research acknowledges that even advanced 4D-DIA technology cannot detect all proteoforms due to their extensive diversity and complexity. Challenges include the inaccurate identification of post-translationally modified proteins or those with unique structural features. Current methods may fail to fully characterize splice variants, degradation products, or modified forms, leading to incomplete/inaccurate proteomic analyses. Future studies should prioritize proteoform analysis, as these variants likely play critical roles in reproduction, such as in sperm genesis, maturation, capacitation, and fertilization. This enhances understanding of equine evolutionary trajectories and informs genetic breeding programs using interspecies genetic variations.

The metabolomic analysis of seminal plasma from light and draft stallions identified 56 molecules, of which 11 metabolites exhibited significant concentration differences [48]. This highlights metabolomic fingerprints distinguishing the two horse types. Similarly, our study of horse and donkey seminal plasma revealed distinct metabolites enriched in the starch/sucrose metabolism, pantothenate/CoA biosynthesis, and tyrosine metabolism pathways. Glucose in these pathways may regulate energy production and sperm motility, critical for viability and fertilization [49,50]. Wang’s findings showed α-amylase supplementation improved boar semen quality, supporting its role in sperm health [51]. Pantothenate, a CoA precursor, is essential for lipid metabolism (a key energy source for sperm). Deficiencies may impair motility, morphology, and maturation. Sousa’s ram study linked this pathway to sperm function support [52]. Tyrosine influences sperm production, maturation, and motility via catecholamine (e.g., dopamine) synthesis. Aberrant metabolism may disrupt sperm regulation [53,54,55,56]. Griffin’s stallion study associated tyrosine-related caseins with fertility: caseins were more abundant in high-fertility samples, potentially protecting sperm from harmful proteins [57]. While our study lacked high-/low-fertility comparisons, tyrosine metabolism enrichment in normal equids suggests its role in sperm quality assessment [58]. Therefore, we propose that tyrosine metabolism in the seminal plasma plays a critical role in assessing sperm quality and fertility in equids. However, regarding metabolite analysis, equine seminal plasma harbors a diverse array of metabolites spanning multiple categories, such as amino acids, sugars, lipids, vitamins, organic acids, and others. Given the substantial variability in the physicochemical properties among different metabolite classes, no single extraction protocol can universally accommodate all metabolites. In practical research, reliance on a singular extraction method may lead to suboptimal extraction efficiency or complete failure to recover certain metabolites, consequently omitting critical metabolic information and impeding a holistic understanding of the metabolic profile of equine seminal plasma.

**Figure 4:**
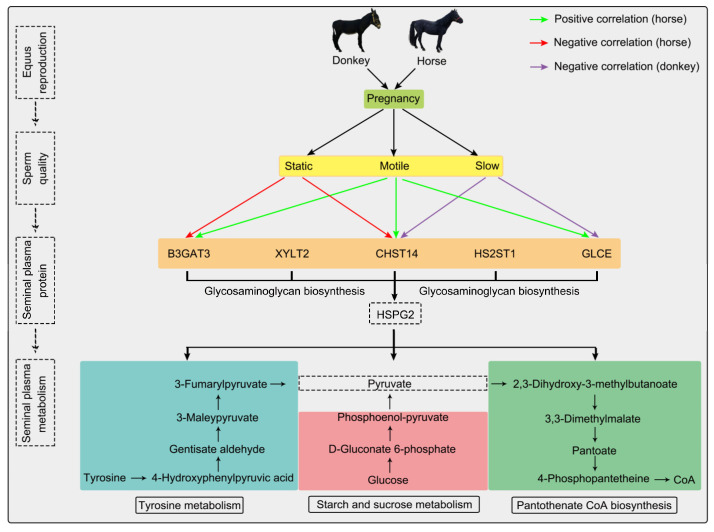
Integrative proteomics and metabolomics analyses of seminal plasma revealed the sperm quality and reproductive capacity of equines. Upon combined proteomic and metabolomic analysis, we discerned several seminal plasma proteins and metabolites intimately associated with sperm quality and fertility in equine species. The green arrow indicates that horse seminal plasma proteins are positively correlated with sperm motility; the red arrow indicates that horse seminal plasma proteins are negatively correlated with sperm motility; the purple arrow indicates that donkey seminal plasma proteins are negatively correlated with sperm motility.

This study conducted proteomic and metabolomic analyses of horse and donkey seminal plasma to identify fertility-associated proteins and pathways. These proteins, namely B3GAT3, XYLT2, CHST14, HS2ST1, GLCE, and HSPG2, interact with one another. Moreover, HSPG2 is connected to the metabolic pathways of starch and sucrose metabolism, pantothenate, CoA biosynthesis, and tyrosine metabolism identified through the metabolomic analysis of seminal plasma, as protein synthesis consumes energy and generates metabolites. Among these metabolic pathways, D-glucose, 4-phosphopantetheine, and 4-hydroxyphenylpyruvic acid are key metabolites, and they are linked via the intermediate pyruvate (Figure 4). These metabolites converge on pyruvate, a glycolytic intermediate, which may indirectly influence sperm function by supporting energy metabolism despite having no established direct causal link to sperm quality [59]. For example, pyruvate’s role in energy supply during sperm motility suggests its potential impact on sperm quality, though further research is needed to clarify this relationship [60,61]. We previously performed a comparative analysis of the seminal plasma proteomes in horses and donkeys, emphasizing species-specific marker proteins to clarify the differences in seminal proteins between these two species [62]. While this study employed the same seminal plasma sequencing samples as the previous analysis, we further integrated metabolomics and multiomics comprehensive analyses, correlating these findings with sperm quality to address the scientific question of “How do proteins and metabolites jointly influence sperm quality?”. Although the two manuscripts are complementary, they differ significantly in their focus and contribution. Consequently, this research not only broadens the research dimension but also offers valuable insights into the reproductive biology of equids (horses and donkeys).

## 5. Conclusions

Through comprehensive analysis of seminal plasma proteomics and metabolomics, we deepened our understanding of sperm quality and fertility in equine species. Proteomics identified seminal plasma proteins associated with sperm motility, while metabolomics revealed key metabolites linked to sperm function, such as D-glucose and 4-phosphopantetheine. By integrating both analyses, we identified several potential proteins and metabolites correlated with sperm quality and reproductive capacity in equine species. Key proteins included B3GAT3, XYLT2, CHST14, HS2ST1, GLCE, and HSPG2; key metabolites included D-glucose, 4-phosphopantetheine, and 4-hydroxyphenylpyruvic acid. This integrated analysis revealed the molecular mechanisms underlying sperm quality and fertility in equine species. Detecting these biomarkers in seminal plasma enables the more accurate evaluation of sperm quality and provides scientific evidence for equine reproductive management. In the future, we aim to develop more precise and convenient detection methods to support equine reproductive health.

## Figures and Tables

**Table 1:** Sperm quality of horse and donkey.

Project	Parameter	Horse	Donkey
Sperm motility	Static	31.64 ± 6.62	9.90 ± 0.63 **
	Progressive	35.07 ± 1.76	71.57 ± 6.21 **
	Motile	68.37 ± 6.62	88.77 ± 2.92 **
	Slow	12.17 ± 4.68	1.77 ± 0.71 *
Sperm morph	Normal morph	77.14 ± 15.76	91.87 ± 7.70
	Bent tail	4.47 ± 2.25	2.64 ± 0.93
	Coiled tail	0.90 ± 0.63	0.74 ± 0.71

Categorical variables were compared via the chi-square test, with the horse and donkey sperm being compared separately. A single asterisk (*) indicates a statistically significant difference (*p* < 0.05), and two asterisks (**) indicate a statistically significant difference (*p* < 0.01).

**Table 2:** Reproduction ability of horse and donkey.

Project	Species	Number of Matings	Number of Pregnancies	Pregnancy Rate
Reproductive ability	Horse	37	25	67.57%
		29	21	72.42%
		35	23	65.72%
	Donkey	6	4	66.67%
		40	30	75.00%
		30	23	76.67%

Categorical variables were compared via the chi-square test for horse and donkey reproduction ability.

## Data Availability

All data are obtainable in the article and its Supplementary Materials file or upon request addressed to the authors.

## Supplementary Materials

The following supporting information can be downloaded at https://www.mdpi.com/article/10.3390/proteomes13030033/s1. Figure S1: The abundance of specific proteins was quantified in the seminal plasma of both horses and donkeys; Figure S2: The negative ion mode in the seminal plasma metabolome of horses and donkeys; Figure S3: The data on seminal plasma proteins and metabolites of horses and donkeys.
