# Supplementary material for: Comprehensive Integrated Analyses of Proteins and Metabolites in Equine Seminal Plasma (Horses and Donkeys)
Source: Proteomes. 2025 Jul 4;13(3):33. doi: 10.3390/proteomes13030033 (PMC12285962; doi:10.3390/proteomes13030033)
Supplement: Supplementary file 1 [file proteomes-13-00033-s001.zip › proteomes-3620347-supplementary.pdf]

proteomes

Article

Comprehensive Integrated Analyses of Proteins and Metabolites in Equine Seminal Plasma (Horses and Donkeys)

Xin Wen †, Gerelchimeg Bou †, Qianqian He, Qi Liu, Minna Yi, and Hong Ren *

Inner Mongolia Key Laboratory of Equine Science Research and Technology Innovation, Inner Mongolia Agricultural University, Hohhot, 010018, China; wenxin618@imau.edu.cn (X.W.); gerelchimeg@imau.edu.cn (G.B.); qianqianhe202309@163.com (Q.H.); liuqi99967@163.com (Q.L.); yiminna2020@163.com (M.Y.)

* Correspondence: renhong1980@126.com

† These authors contributed equally to this work.

Supplementary Materials

Supplementary Figure 1

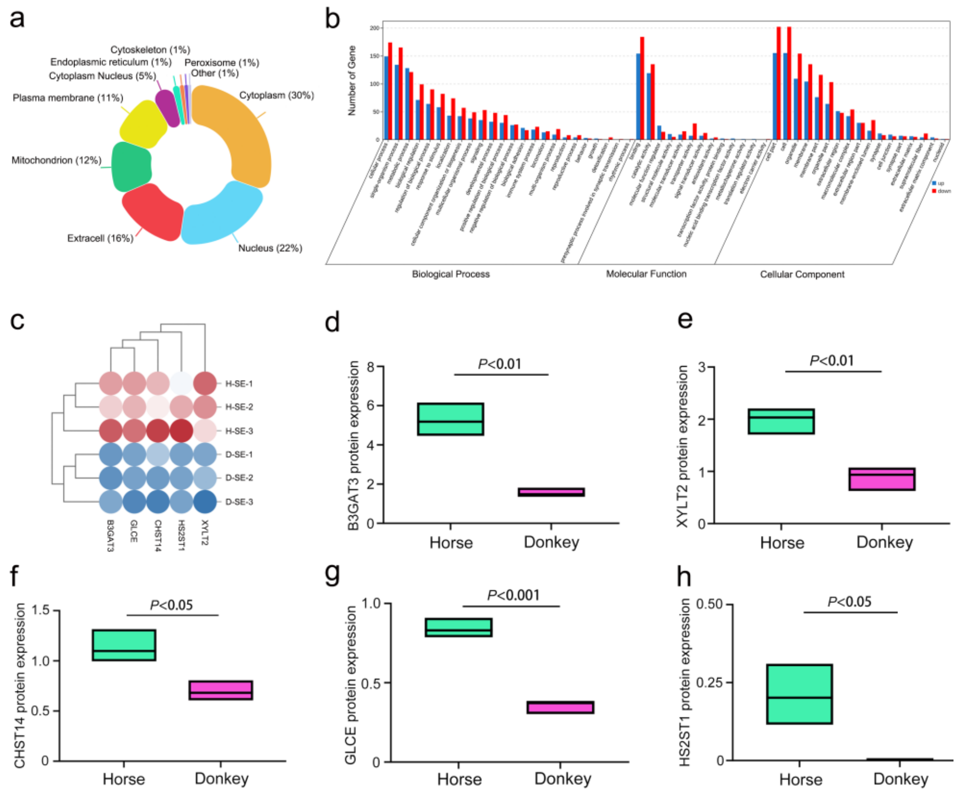

Figure S1. The abundance of specific proteins was quantified in the seminal plasma of both horses and donkeys. (a) Circle diagram revealing the subcellular localization of proteins in the seminal plasma of horses and donkeys. **(b)** Bar chart exhibiting the results of the GO enrichment analysis of DEPs in the seminal plasma of horses and donkeys, including biological processes, molecular functions, and cellular components. **(c)** Heatmap showing all the DEPs in the glycosaminoglycan biosynthesis signaling pathway. **(d)** B3GAT3 protein expression in the seminal plasma of horses and donkeys, determined through proteomic sequencing data. **(e)** Expression of the XYLT2 protein in the seminal plasma of horses and donkeys. **(f)** Expression of the CHST14 protein in the seminal plasma of horses and donkeys. **(g)** Expression of the GLCE protein in the seminal plasma of horses and donkeys. **(h)** Expression of the HS2ST1 protein in the seminal plasma of horses and donkeys. The d, e, f, g, and h data originate from the proteomic sequencing data of the seminal plasma in horses and donkeys. The data are presented as the means ± SDs. $P < 0.05$ was considered to indicate a statistically significant difference.

Supplementary Figure 2

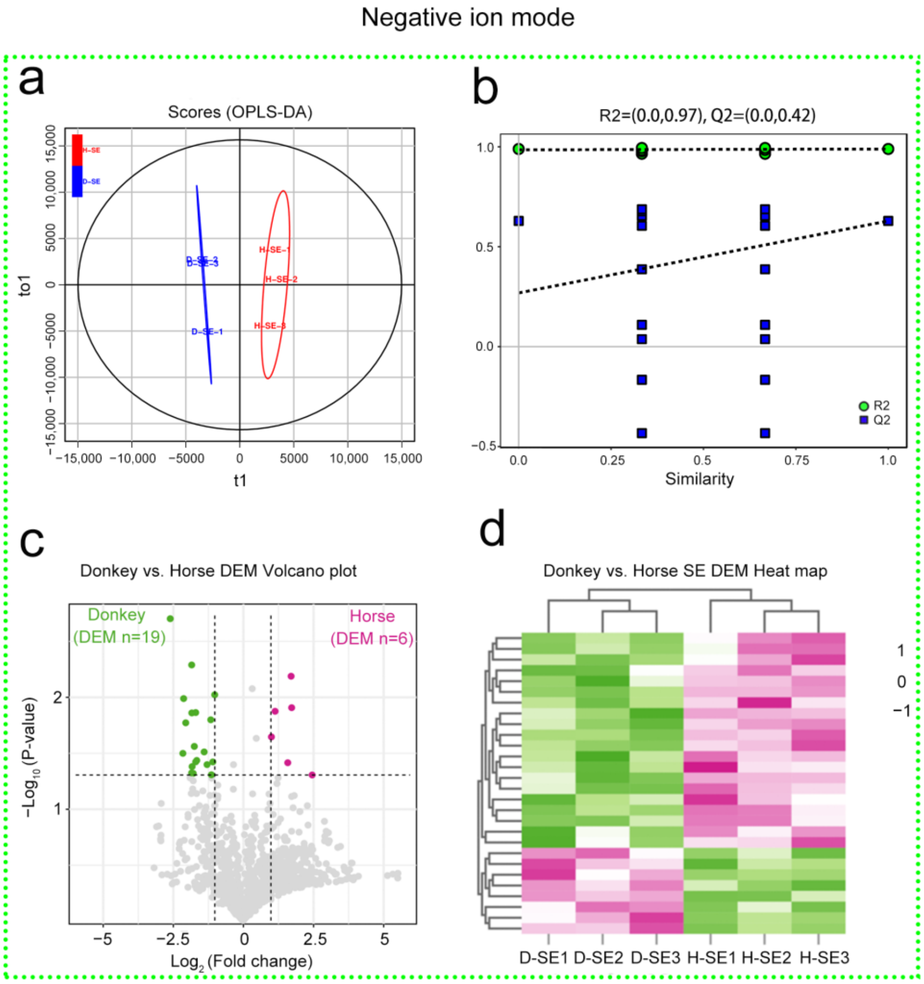

Figure S2. The negative ion mode in the seminal plasma metabolome of horses and donkeys. **(a)** OPLS–DA of metabolomic data for seminal plasma of donkeys and horses in positive ion mode. **(b)** Diagram of model verification in positive ion mode. **(c)** Volcano plot of DEMs between the seminal plasma of a donkey and a horse in positive ion mode. **(d)** Heatmap of metabolites between the seminal plasma of a donkey and a horse in negative ion mode.

Supplementary Figure 3

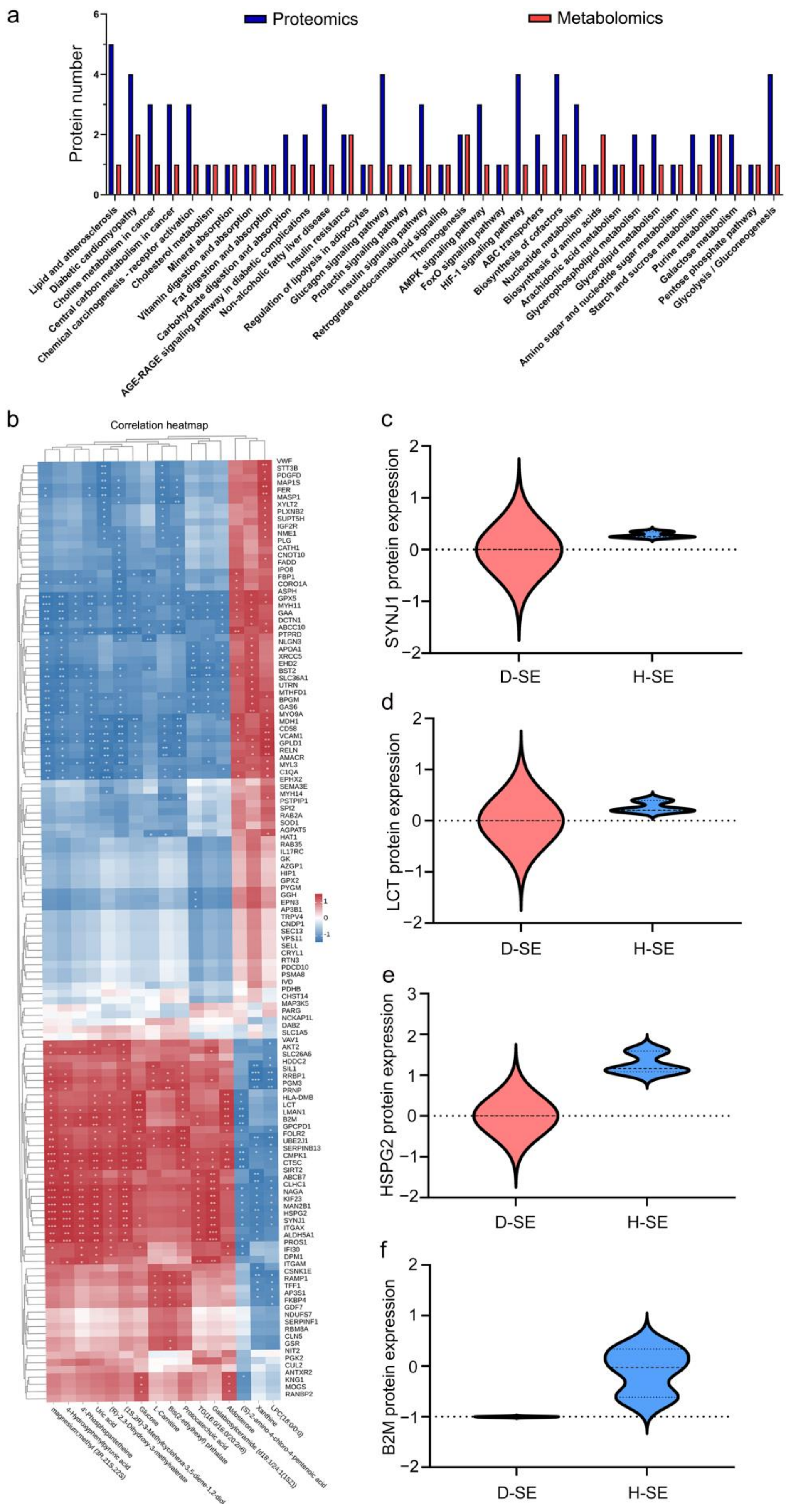

Figure S3. The data on seminal plasma proteins and metabolites of horses and donkeys. (a) Bar chart presenting the 36 KEGG signaling pathways identified by both proteomic and metabolomic analyses. **(b)** Heatmap illustrating the correlations among all proteins and metabolites within the shared signaling pathways. **(c)** Expression of the SYNJ1 protein in the seminal plasma of horses and donkeys. **(d)** Expression of the LCT protein in the seminal plasma of horses and donkeys. **(e)** The expression of HSPG2 protein in the seminal plasma of horses and donkeys. **(f)** Expression of the B2M protein in the seminal plasma of horses and donkeys.
